# Protocol for the “Chemobrain in Motion – study” (CIM – study): a randomized placebo-controlled trial of the impact of a high-intensity interval endurance training on cancer related cognitive impairments in women with breast cancer receiving first-line chemotherapy

**DOI:** 10.1186/s12885-018-4992-3

**Published:** 2018-11-06

**Authors:** Max Oberste, Nils Schaffrath, Katharina Schmidt, Wilhelm Bloch, Elke Jäger, Karen Steindorf, Philipp Hartig, Niklas Joisten, Philipp Zimmer

**Affiliations:** 1 Department of Molecular and Cellular Sport Medicine, Institute of Cardiovascular Research and Sports Medicine, German Sport University Cologne, Am Sportpark Müngersdorf 6, 50933 Cologne, Germany; 2 Department for Oncology and Hematology, Clinic Northwest, Steinbacher Hohl 2-26, 60488 Frankfurt am Main, Germany; 3 Division of Physical Activity, Prevention and Cancer, German Cancer Research Center (DKFZ), Im Neuenheimer Feld 581, 69120 Heidelberg, Germany

**Keywords:** Cancer, Cognition, Exercise, Breast cancer, Chemobrain, High-intensity interval endurance training

## Abstract

**Background:**

Up to 80% of breast cancer patients suffer from Cancer Related Cognitive Impairments (CRCI). Exercise is suggested as a potential supportive care option to reduce cognitive decline in cancer patients. This study will investigate the effects of a high-intensity interval endurance training (HIIT) on CRCI in breast cancer patients. Potentially underlying immunological and neurobiological mechanisms, as well as effects on patients’ self-perceived cognitive functioning and common cancer related side-effects, will be explored.

**Methods:**

A single-blinded randomized controlled trial will be carried out. The impact of HIIT on CRCI will be compared to that of a placebo-intervention (supervised myofascial release training). Both interventions will be conducted simultaneously with the patients’ first-line chemotherapy treatment typically lasting 12–18 weeks. Fifty-nine women with breast cancer will be included in each of the two groups. The study is powered to detect (α = .05, β = .2) a medium effect size difference between the two groups (d = .5) in terms of patients’ change in cognitive testing performances, from baseline until the end of the exercise-intervention. The cognitive test battery, recommended by the International Cancer and Cognition Task Force to assess CRCI, will be used as primary measure. This includes the Hopkins Verbal Learning Test (learning/verbal memory), the Controlled Oral Word Association Test (verbal fluency) and the Trail-Making-Test A/B (attention/set-switching). The following endpoints will be assessed as secondary measures: Go-/No-Go test performance (response inhibition), self-perceived cognitive functioning, serum levels of pro- and antiinflammatory markers (tumor necrosis factor alpha, Interleukin-6, Interleukin-1 alpha, Interleukin-1 beta, C-reactive protein, Interleukin-1 receptor antagonist and Interleukin-10), serum levels of neurotrophic and growth factors (brain-derived neurotrophic factor, insulin-like growth factor 1 and vascular endothelial growth factor), as well as common cancer-related side effects (decrease in physical capacity, fatigue, anxiety and depression, sleep disturbances, quality of life and chemotherapy compliance).

**Discussion:**

This study will provide data on the question whether HIIT is an effective supportive therapy that alleviates CRCI in breast cancer patients. Moreover, the present study will help shed light on the underlying mechanisms of potential CRCI improving effects of exercise in breast cancer patients.

**Trial registration:**

DRKS.de, German Clinical Trials Register (DRKS), ID: DRKS00011390, Registered on 17 January 2018.

**Electronic supplementary material:**

The online version of this article (10.1186/s12885-018-4992-3) contains supplementary material, which is available to authorized users.

## Background

Up to 80% of breast cancer patients demonstrate a decrease in their cognitive capacity [1–5]. Most frequently reported are mild to moderate deficits in processing speed, attention, memory and executive functions (1–3, 5). In about 35% of affected patients, cognitive symptoms persist for months, or even years, after completion of medical treatments [6, 7], impairing daily functioning [8], limiting ability to return to work [9] and decreasing overall quality of life [8, 10–12]. Cognitive decline in breast cancer patients is mostly attributed to chemotherapy [13–15] leading to the expression of “Chemobrain” [16]. However, recent research shows that other factors, besides chemotherapy, can adversely impact cognition of breast cancer patients [17–23]. Consequently, the term “Cancer Related Cognitive Impairment” (CRCI) is currently preferred in the scientific literature [24, 25].

The underlying mechanisms of CRCI are still not fully understood. Recent findings indicate a key role of inflammatory pathways in the genesis of CRCI [26]. Affected patients show increased levels of proinflammatory markers, such as tumor necrosis factor alpha (TNF-α), Interleukin-6 (IL-6), Interleukin-1 Alpha (IL-1α), Interleukin-1 Beta (IL-1β) and C-reactive protein (CRP) [27–30]. At the same time, antiinflammatory cytokines, such as Interleukin-1 receptor antagonist (IL-1RA) and Interleukin-10 (IL-10) are decreased [30]. Hypothesized mechanisms of CRCI also include decreased levels of neurotrophic and growth factors, such as brain-derived neurotrophic factor (BDNF), insulin-like growth factor 1 (IGF-1) and vascular endothelial growth factor (VEGF) [31–35].

Research on therapeutic interventions alleviating CRCI in breast cancer patients is still scarce [25]. Recently, exercise increasingly gains attention as a potential supportive care option to reduce CRCI [24, 25, 36, 37]. In healthy elderly adults [38–40] and patients suffering from neurodegenerative diseases [41–43], there is substantial evidence that underlines the benefits of aerobic endurance training for functioning and structure of the central nervous system (CNS). However, initial studies, which are testing the effects of endurance exercise programs on CRCI in patients with breast cancer, show only small beneficial effects, if at all [44–48]. One reason could be that the applied training regimens, with low to moderate exercise intensities, were not intense enough to have meaningful effects on patients’ cognition. Moreover, recent research, in patients with breast cancer, shows that high-intensity endurance training is not only safe and feasible [49, 50], but is also more efficient than low to moderate endurance exercise, in terms of cognitive benefits [51]. The assumption, that higher intense exercise regimens have superior beneficial effects on CRCI, is supported by research demonstrating a positive dose-response relationship among exercise intensity and exercise induced anti-inflammatory effects/neurotrophin expression [52–54].

High-intensity interval endurance training (HIIT) seems to be an especially promising and time-efficient regimen, as potential supportive care option for CRCI, among the more intense exercise interventions. High-intensity interval endurance training increases peak aerobic fitness, it alleviates cardiovascular risk and fatigue in cancer patients [55, 56]. All the aforementioned are associated with CRCI [24]. HIIT increases anti-inflammatory markers [57] and neurotrophic factors [58], in a particularly effective and efficient way.

The main objective of this study will be to investigate the effects of a HIIT program on CRCI in breast cancer patients undergoing first-line chemotherapy, compared to a placebo-control group. The cognitive test battery, recommended by the International Cancer and Cognition Task Force to assess CRCI, will be used as primary measure [59]. The following endpoints will be assessed as secondary measures: Go-/No-Go-test performance (response inhibition), self-perceived cognitive performance, serum levels of pro- and antiinflammatory markers (TNF-α, IL-6, IL-1α, IL-1β, CRP, IL-1RA, IL-10), serum levels of neurotrophic and growth factors (BDNF, IGF-1, VEGF) as well as common cancer-related side effects (decrease in physical capacity, fatigue, anxiety and depression, sleep disturbances, quality of life and chemotherapy compliance).

## Methods

**Fig. 1:**
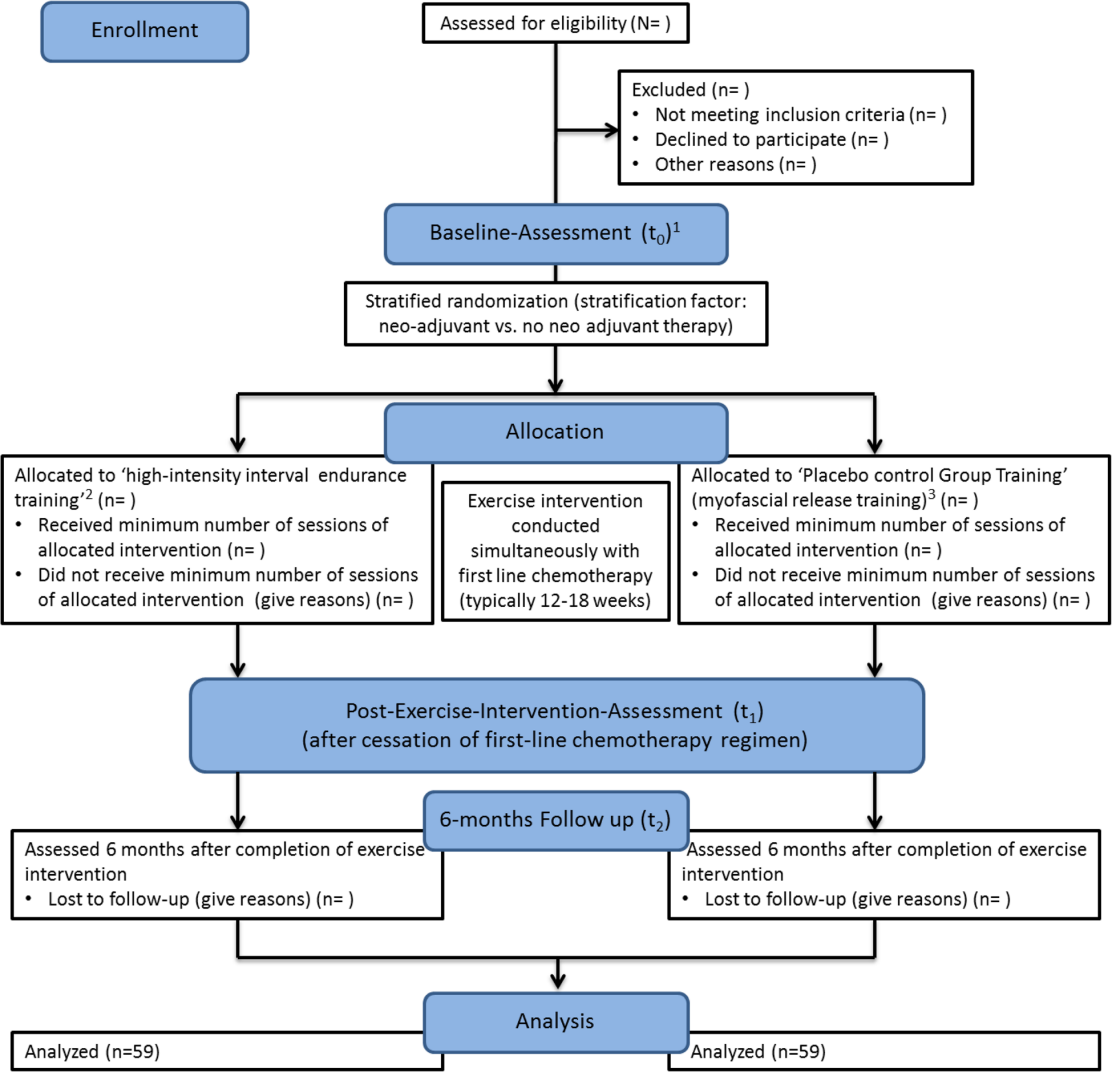
Flowchart of the trial. (1) Baseline measurement will be conducted shortly after diagnosis and 1–3 days before beginning of patients’ medical treatment; (2) Participants allocated to the high-intensity interval endurance training perform 3 × 3 min high-intensive intervals, interspersed by 90 s active rest-intervals during the first 8 weeks of their chemotherapy (duration ≈ 20–25 min). During the second half of participants’ chemotherapy, the number of intervals will be increased to 5 (duration ≈ 30–35 min); (2) Participants allocated to the Placebo Control Group receive a supervised myofascial release training program, inducing hardly any muscular effort and cardiovascular stimulation. Duration of sessions in the Placebo Control Group correspond to the duration of sessions in the high-intensity interval endurance training

The present study is designed as a longitudinal randomized interventional study with two study arms. Participants will be allocated randomly to one of two forms of supervised exercise (see “Groups and interventions” section). Exercise will be done simultaneously with patients’ first-line chemotherapy regimen typically lasting 12–18 weeks (parallel group, single-blinded, randomized controlled trial (RCT)). That means that the exercise intervention will stop when chemotherapy stops. The pre-specified objectives and hypotheses are listed in Table 1. The study flowchart is shown in Fig 1. The study protocol was approved by the Ethics Commission of the Legal Department of the Hessen Regional Medical Council (Germany) (reference number: FF175/2016) and is registered at the WHO trial register (ID: DRKS00011390, see also Additional file 1). Any modifications to the protocol which may impact the implementation of the study, or impact any potential patient benefit, or may affect patient safety, including changes of study objectives, study design, patient population, sample sizes, study procedures, or significant administrative aspects, will require a formal amendment at the Ethics Commission. The project adheres to the declaration of Helsinki.

**Table 1:** In- and exclusion criteria

Inclusion criteria	Exclusion criteria
• Initial diagnosis of mammary carcinoma stage I-IIIA • Female • Pending chemotherapy (neoadjuvant or adjuvant) • No contraindication to high-intense exercise	• Age < 18 years • History of former cancer diagnoses • Coronary Heart disease • Heart failure • Advanced lung disease • Major psychiatric disorders • Neurodegenerative disease • Cognitive dysfunction • Incapable to speak or understand German

### Study population and recruitment

Participants will be recruited at the Department for Oncology and Hematology of the Clinic Northwest and at the Department for Gynecology at the University Clinic in Frankfurt (Germany). Patients with initial diagnosis of mammary carcinoma stage I-IIIA and scheduled chemotherapy, not fulfilling any of the listed exclusion criteria in Table 2, will be recommended for study participation by the attending physician to the principal investigator. Patients will receive detailed descriptions of the study and will be given sufficient time to ask any questions. Patients will be included in the planned study only after they will give written informed consent. The participants will be included chronologically.

**Table 2:** Objectives and hypotheses of the study

Objectives	Hypotheses
Main objective • To evaluate the effects of a HIIT versus placebo, conducted simultaneously with first-line chemotherapy regimen on CRCI, in women with breast cancer	Main hypothesis • In women with breast cancer, the decline in HVLT-R-, TMT-A/B- and COWAT test performance from baseline to completion of the exercise-intervention, is significantly smaller in the HIIT group compared to the control group (hypothesis A)
Secondary objective • To evaluate the effects of a HIIT versus placebo, conducted simultaneously with first-line chemotherapy regimen on self-perceived cognitive performance, in women with breast cancer	Secondary hypotheses • In women with breast cancer, the decline of FACT-COG scores from baseline to completion of the exercise-intervention/to 6-months follow-up^a^_,_ is significantly smaller in the HIIT group compared to the control group (hypothesis B)
• To evaluate the effects of a HIIT versus placebo, conducted simultaneously with first-line chemotherapy regimen on executive functioning subdomain response inhibition, in women with breast cancer	• In women with breast cancer, the decline of INHIB performance from baseline to completion of the exercise-intervention/to 6-months follow up, is significantly smaller in the HIIT group compared to the control group (hypothesis C)
• To evaluate the effects of a HIIT versus placebo, conducted simultaneously with first-line chemotherapy regimen on proinflammatory marker expression, in women with breast cancer	• In women with breast cancer, the increase of serum levels of TNF-α, IL-6, IL-1α, IL-1β, CRP from baseline to completion of the exercise-intervention/to 6-months follow up, is significantly smaller in the HIIT group compared to the control group (hypothesis D)
• To evaluate the effects of a HIIT versus placebo, conducted simultaneously with first-line chemotherapy regimen on antiinflammatory marker expression, in women with breast cancer	• In women with breast cancer, the decrease of serum levels of IL-1RA, IL-10 from baseline to completion of the exercise-intervention/to 6-months follow up, is significantly smaller in the HIIT group compared to the control group (hypothesis E)
• To evaluate the effects of a HIIT versus placebo, conducted simultaneously with first-line chemotherapy regimen on neurotrophin expression, in women with breast cancer	• In women with breast cancer, the decrease of BDNF serum level from baseline to completion of the exercise-intervention/to 6-months follow up, is significantly smaller in the HIIT group compared to the control group (hypothesis F)
• To evaluate the effects of a HIIT versus placebo, conducted simultaneously with first-line chemotherapy regimen on growth factor expression, in women with breast cancer	• In women with breast cancer, the decrease of VEGF and IGF-1 serum levels from baseline to completion of the exercise-intervention/to 6-months follow up, is significantly smaller in the HIIT group compared to the control group (hypothesis G)
• To evaluate the effects of a HIIT versus placebo, conducted simultaneously with first-line chemotherapy regimen on physical fitness, in women with breast cancer	• In women with breast cancer, the decrease of watts per kilogram and VO2_peak_ measured during IXT from baseline to completion of the exercise-intervention/to 6-months follow up, is significantly smaller in the HIIT group compared to the control group (hypothesis H)
• To evaluate the effects of a HIIT versus placebo, conducted simultaneously with first-line chemotherapy regimen on anxiety, fatigue, quality of life, sleep disturbances and chemotherapy compliance, in women with breast cancer	• In women with breast cancer, the change of HADS-D, MFI-20, EORTC-QLQ-C30, QOL-BR23 and PSQI scores from baseline to completion of the exercise-intervention/to 6-months follow up, is significantly more favorable in the HIIT group compared to the control group (hypothesis I)
• To evaluate the sustainability of the effects of a HIIT versus placebo conducted simultaneously with first-line chemotherapy regimen on CRCI in women with breast cancer	• In women with breast cancer, the decline in HVLT-R-, TMT-A/B- and COWAT test performance from baseline to 6-months follow up is significantly smaller in the HIIT group compared to the control group (hypothesis J)
Exploratory mediation analysis	
• To evaluate if the change in proinflammatory marker expression, from baseline to completion of the exercise-intervention, partially mediates the effect of the exercise intervention on the change of cognitive performance, from baseline to completion of the exercise-intervention, in women with breast cancer.	• In women with breast cancer, there is a significant indirect effect of exercise intervention on the change of HVLT-R−/TMT-A/B-/COWAT test performance, from baseline to completion of the exercise-intervention, through the change of TNF-α/IL-6/IL-1α/IL-1β/CRP serum level, from baseline to completion of the exercise-intervention (hypothesis K)
• To evaluate if the change in antiinflammatory marker expression, from baseline to completion of the exercise-intervention, partially mediates the effect of the exercise intervention on the change of cognitive performance, from baseline to completion of the exercise-intervention, in women with breast cancer.	• In women with breast cancer, there is a significant indirect effect of exercise intervention on the change of HVLT-R−/TMT-A/B-/COWAT test performance, from baseline to completion of the exercise-intervention, through the change of IL-1RA/IL-10 serum level, from baseline to completion of the exercise-intervention (hypothesis L)
• To evaluate if the change in neurotrophin marker expression, from baseline to completion of the exercise-intervention, partially mediates the effect of the exercise intervention on the change of cognitive performance, from baseline to completion of the exercise-intervention, in women with breast cancer.	• In women with breast cancer, there is a significant indirect effect of exercise intervention on the change of HVLT-R−/TMT-A/B-/COWAT test performance, from baseline to completion of the exercise-intervention, through the change of BDNF serum level, from baseline to completion of the exercise-intervention (hypothesis M)
• To evaluate if the change in growth factor marker expression, from baseline to completion of the exercise-intervention, partially mediates the effect of the exercise intervention on the change of cognitive performance, from baseline to completion of the exercise-intervention, in women with breast cancer.	• In women with breast cancer, there is a significant indirect effect of exercise intervention on the change of HVLT-R−/TMT-A/B-/COWAT test performance, from baseline to completion of the exercise-intervention, through the change of VEGF/IGF-1 serum level, from baseline to completion of the exercise-intervention (hypothesis N)

^a^ 6-month follow up will be conducted 6 months after completion of the exercise intervention

*HIIT* high-intensity interval endurance training, *HVLT-R* Hopkins Verbal Learning Test - Revised, *COWAT* Controlled Oral Word Association Test, *TMT-A/B* Trail Making Test part A/part B, *INHIB* Response Inhibition Test*, FACT-COG* Functional Assessment of Cancer Therapy – Cognitive function, *TNF-α* tumor necrosis factor alpha, *IL-6* Interleukin-6, *IL-1α* Interleukin-1 Alpha, *IL-1β* Interleukin-1 Beta, *CRP* C-reactive protein*, IL-1RA* Interleukin-1 receptor antagonist, *IL-10* Interleukin-10, *BDNF* brain-derived neurotrophic factor, *VEGF* vascular endothelial growth factor, *IGF-1* insulin-like growth factor 1, *VO2*_*peak*_ peak of oxygen uptake in milliliter oxygen uptake per kilogram bodyweight per minute, *IXT* incremental exercise test, *HADS-D* German version of the Hospital Anxiety and Depression Scale *MFI-20* The multidimensional Fatigue Inventory, *EORTC-QLQ-C30* core questionnaire 30 items of the European Organization for Research and Treatment of Cancer, *QOL-BR23* the breast cancer specific quality of life questionnaire of the EORTC *PSQI* Pittsburgh Sleeping Quality Index

### Randomization and blinding

Eligible patients will be randomly allocated to one of two experimental groups (see “Groups and treatments” section below). A stratified block randomization with permuted block length will be conducted, using the “Randomization-In-Treatment-Arms” software (RITA, Evident, Germany). The application of neo-adjuvant chemotherapy (neo-adjuvant vs. no neo-adjuvant treatment) will be the stratification factor.

In the present study, participants will be kept blind to the research question, as much as possible. They will be informed that two exercise interventions (HIIT vs. myofascial release training) will be compared, in terms of their effect on cognitive performance, blood parameters and common cancer related side effects, in patients with breast cancer. Accordingly, patients will not know the specific hypotheses of the trial. They will not know if they are allocated to the experimental or to the control group.

### Groups and interventions

Patients will be allocated either into a HIIT or a placebo control group. High-intensity interval endurance training and placebo control group will be conducted at the Clinic Northwest in Frankfurt (Germany), simultaneously with the patients’ first-line chemotherapy regimen, typically lasting 12 to 18 weeks.

- HIIT Group
  Patients will receive a supervised HIIT on a stationary bicycle ergometer (Ergoselect 100 Typ K, Ergoline, Bitz, Germany). Sessions will be carried out 3 times per week with at least 24 h rest between sessions. The first 5 min (min) of each training session will be warm-up at low-intensity (cadence at 60–70 rpm (rpm) and wattage according to 57–63% of patient’s maximum heart rate (HR_max_)). During the first 8 weeks of patients’ chemotherapy, HIIT will consist of 3 × 3 min high-intensive intervals (cadence at 80–100 rpm and wattage according to 85–90% of HR_max_) interspersed by 90 s active rest-intervals (cadence at 60–70 rpm and wattage according to 57–63% of HR_max_). During the second half of patients’ chemotherapy, the number of intervals will be increased to 5 [49]. The last 5 min of each training session will be cool-down at low-intensity (cadence at 60–70 rpm and wattage according to 57–63% of HR_max_). Accordingly, duration of each HIIT session will be approximately 20–25 min during the first 8 weeks of patients’ chemotherapy and 30–35 min during the second half of patients’ chemotherapy. In cases of participants showing blunted heart rate response to exercise, (i.e. resulting from medications like beta-blocker medication) ratings of perceived exertion on the Borg scale [60] will be used to control exercise intensity rather than heart rate [61]. In such cases, warm up, cool down and active rest intervals, interspersed between high-intensive intervals, will be conducted at wattage in accordance with a rating of perceived exertion of 10–12 on the Borg scale. High-intensive intervals, in such cases, will be conducted at wattage in accordance with a rating of perceived exertion of 16–18 on the Borg scale. Any deviations from the protocol will be recorded. High-intensity interval endurance training will be conducted in addition to the standard medical treatment and physiotherapy.
- Placebo Control Group (PCG)
  Patients allocated to the PCG will receive a supervised myofascial release training 3 times per week with at least 24 h rest between sessions. The training sessions consist of seven standardized exercises using a foam roll (Blackroll, Bottighofen, Swiss): self-massage of the soles of the feet (standing on one leg), of the calves (lying in dorsal position), of the hamstrings (lying in dorsal position), of the thighs (manually while lying in dorsal position), of the neck (lying in dorsal position), of the lower and upper back (leaning backwards against a wall in standing position), as well as self-massage of the upper arm and shoulder (leaning sideways against a wall in standing position). During the first 8 weeks of patients’ chemotherapy, each exercise will be repeated 6 times on each side of the body. During the second half of patients’ chemotherapy, each exercise will be repeated 10 times on each side of the body. Between each exercise, participants will do low-intense shoulder and hip mobilization exercises for 2 min. Accordingly, duration of each PCG session will be approximately 20–25 min during the first 8 weeks of patients’ chemotherapy and 30–35 min during the second half of patients’ chemotherapy. Any deviations to this protocol will be recorded. Myofascial release training will also be conducted in addition to the standard medical treatment and physiotherapy.

PCG-training was designed as attention control condition. Both treatments, HIIT and PCG-training, will be carried out individually and will be supervised by independent sport-therapists. Training frequency and duration of HIIT and PCG training sessions will be equal, to ensure that the attention, the treatment contact and the nonspecific therapist effects are balanced between HIIT group and PCG. However, PCG-training (myofascial release training), unlike the HIIT, will induce hardly any muscular effort and cardiovascular stimulation [62].

Exercise-treatments will be carried out neither on chemotherapy days, nor 24 h after a chemotherapy application [63]. Other contraindications that prohibit participation in a training session of HIIT or PCG are described below:

- Infections
- Hemoglobin < 8 g per deciliter
- Platelet counts < 10,000/μl
- Fever
- Nausea
- Dizziness

Patients’ compliance to the exercise program will be strengthened by the individual supervision patients receive. Each patient will have one sport-therapist that will run all the training sessions. The sport-therapist will monitor the patient’s exercise sessions closely and will motivate the patient to apply the protocol. The sport-therapist will provide feedback on patients’ progress and will discuss benefits and barriers of the exercise with the patient. Feedback and support from sport-therapists have been reported to promote adherence to exercise programs in breast cancer patients [64, 65]. To further increase adherence to the training, participants will receive an E-mail every Friday afternoon by their sport-therapist summarizing their previous workout and reinforcing patients’ adherence by praising their attendance and discipline in the program. The E-mail will also contain the training schedule for the following week with the days and times of the planned training sessions.

### Data collection and timeline

**Fig. 2:**
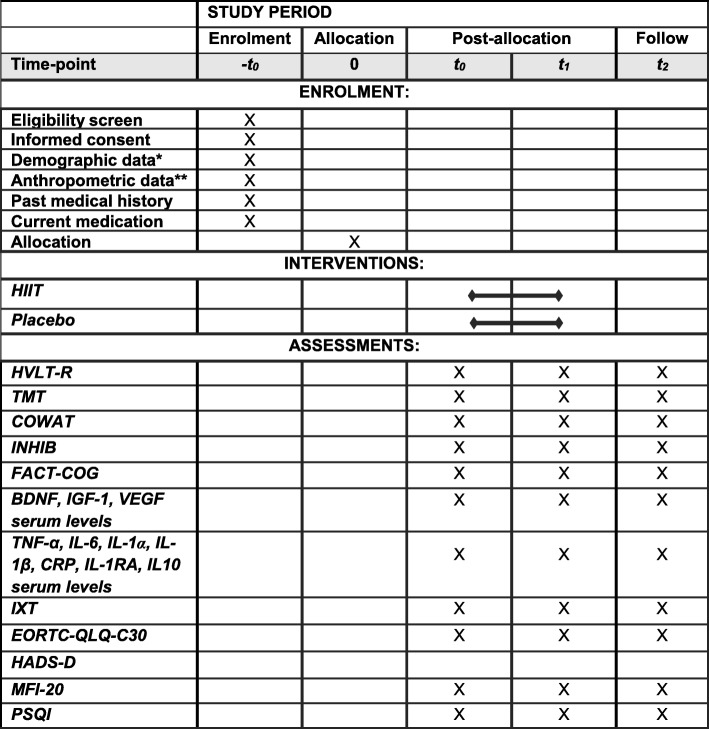
The schedule of enrolment, interventions and assessments. *-t*_*0*_ during the process of enrolment, *t*_*0*_ baseline, *t*_*1*_ after completion of exercise intervention (equivalent to the end of the patients’ first-line chemotherapy), *t*_*2*_ 6 months after completion of exercise intervention, *HIIT* high-intensity interval endurance training; *Placebo* control group training (supervised myofascial release training), *HVLT-R* Hopkins Verbal Learning Test - Revised, *COWAT* Controlled Oral Word Association Test, *TMT* Trail Making Test part A/part B, *INHIB* Response Inhibition Test, *FACT-COG* Functional Assessment of Cancer Therapy – Cognitive function, *TNF-α* tumor necrosis factor alpha, *IL-6* Interleukin-6, *IL-1α* Interleukin-1 Alpha, *IL-1β* Interleukin-1 Beta, *CRP* C-reactive protein*, IL-1RA* Interleukin-1 receptor antagonist, *IL-10* Interleukin-10, *BDNF* brain-derived neurotrophic factor, *VEGF* vascular endothelial growth factor, *IGF-1* insulin-like growth factor 1, *IXT* incremental exercise test, *MFI-20* The multidimensional Fatigue Inventory, *EORTC-QLQ-C30* core questionnaire 30 items of the European Organization for Research and Treatment of Cancer, *HADS-D* German version of the Hospital Anxiety and Depression Scale *PSQI* Pittsburgh Sleeping Quality Index. * As demographic data: age, sex, education, socioeconomic status will be captured. ** As anthropometric data height, weight and BMI will be captured

Data will be collected during the process of enrollment (−t_0_), at baseline (t_0_), after completion of the exercise intervention (equivalent to the end of patients’ first-line chemotherapy) (t_1_) and 6 months after completion of the exercise intervention (follow-up, t_2_). During the enrolment process, the patients’ medical record and planned therapy, demographic (age, sex, education) and anthropometric data (weight, height, Body Mass Index), as well as past medical history and current medication will be captured. Baseline measurement will be conducted shortly after diagnosis and 1–3 days before beginning of patients’ medical treatment. At t_0_, primary outcome measures and all secondary outcome measures of the study (see “Primary endpoint” and “Secondary endpoints” section below), will be captured. Following the intervention period, i.e. the end of first-line chemotherapy (t_1_), and 6 months after that (t_2_), the primary outcome measures and all the secondary outcome measures will be re-assessed. The detailed study schedule is provided in Fig 2. The “Standard Protocol Items: Recommendations for Interventional Trials” (SPIRIT) checklist [66] is provided as an additional file 2 to this publication.

### Primary endpoint

The primary endpoint of the present study will be the change of test-scores from baseline (t_0_) to after completion of the exercise intervention (t_1_), in the test battery recommended by the International Cancer and Cognition Task Force (ICCTF) to capture CRCI [59]. The ICCTF test battery comprises of the Hopkins Verbal Learning Test – Revised (HVLT-R), Trail-Making-Test A/B (TMT-A/TMT-B) and the Controlled Oral Word Association Test (COWAT).

- The HVLT-R
  The HVLT-R was developed to measure forgetfulness in the domain of verbal memory in individuals at the age of 16 and older [67]. The test includes a list of 12 nouns, which will be read to the participants. Then, the participants will be instructed to repeat as many words from that list as they can remember in any order. This process will be repeated three times. The sum of correct recalled words at all 3 learning trials (“Total-Recall”) has been described as measure of learning efficiency and short-term verbal memory [68] and will be used for analyses. The HVLT-R offers six parallel forms. As recommended for repeated testing, the parallel forms 1, 2 and 4 will be used at t_0_, t_1_ and t_2_ [68]. The order of test forms at the 3 measurement time points will be random but counterbalanced across participants. The HVLT-R shows adequate psychometric properties [69, 70].
- TMT-A/B
  The TMT-A will instruct participants to connect randomly distributed numbers from 1 to 25 as quickly and as correctly as possible, in ascending order. The time participants require for completing TMT part A has been described as a valid measure of selective attention [71] and will be used for analyses in the planned study. TMT-B will instruct participants to connect randomly distributed numbers (1 to 13) and letters (A to L) in ascending order, alternating between numbers and letters as quickly and as correctly as possible. The time participants require for completing TMT part B has been reported as valid measure of executive functioning subdomain set-shifting [71] and will be used for analyses. In the present study, the Langensteinbacher version of the TMT (TMT-L) [72] will be applied. It will be operated via the Wiener Test-System (WTS) (Schuhfried, Vienna, Austria) and adequate psychometric properties have been reported for it [72]. TMT-L parallel versions S1, S2 and S3 will be used in a random order at the 3 measurement time-points, but counterbalanced across participants.
- COWAT
  The German version of the COWAT will be used in the present study [73]. Participants will be instructed to name as many words as possible in one min, beginning with a given letter. As recommended in the German test-manual [73], the letters S, P and M will be used at the 3 measurement time points. The order of the letters will be random, but counterbalanced across participants. The number of words participants will produce will be used for analyzation. Adequate psychometric properties for the German version of the COWAT have been reported [73].

### Secondary endpoints

The following secondary endpoints will be assessed in the present study:

- Go-/No-Go-test performance
  The Response Inhibition Test (INHIB) [74] will be applied as additional cognitive test in the planned study. It will also be operated via the WTS. The INHIB is a classical Go-/No-go test paradigm. Participants will be presented with Go- and No-Go-stimuli. They will be instructed to push a button, as quickly as possible, as a response to a Go-stimulus. The vast majority of stimuli will be Go-stimuli, forming a response tendency of pushing the button. The number of erroneous reactions to No-go-stimuli has been reported as valid measure of executive functioning subdomain response inhibition [71] and will be used for analyses. Adequate psychometric properties have been reported for the INIHIB [75].
- Self-perceived cognitive performance
  Self-perceived cognitive performance will be assessed using the FACT-COG questionnaire (German Version 3) [76], which was developed as part of the Functional Assessment of Cancer Therapy-Measurement System [77]. The FACT-Cog version 3 is comprised of 37 items, with 4 subscales: patients’ perceived cognitive impairments, deficits observed or commented by others, perceived cognitive abilities, impact of cognitive changes on quality of life. Respondents will indicate the frequency of each occurrence over the 7 days preceding the test, using a 5-point Likert-type scale ranging from 0 (never) to 4 (several times). For the FACT-Cog adequate psychometric properties have been reported [78].
- Inflammatory marker, neurotrophin and growth factor expression
  Serum levels of TNF-α, IL-6, IL-1α, IL-1β, CRP, IL-1RA and IL-10 as well as of BDNF, IGF-1 and VEGF will be quantified by using enzyme-linked immunosorbent assay (ELISA). Drawing of 8.5 ml venous blood at resting state will be performed at t_0_, t_1_ and t_2_. Samples will be taken from a peripheral vein of the arm and collected in serum gel tubes (S-Monovette® Serum, Sarstedt, Nümbrecht, Germany). After complete coagulation at 4 °C, samples will be centrifuged for 10 min at 1000 g and 4 °C. The serum will be prepared and aliquoted within 24 h after blood drawing and duplicate samples will be stored at − 80 °C until analyzed when samples of all included participants will be collected.
- Patients’ physical fitness
  Patients’ physical fitness will be assessed using an incremental exercise test (IXT) on a bicycle ergometer (Ergoselect 100 Typ K, Ergoline, Bitz, Germany). According to recommended guideline [79], a quasi-ramp protocol will be used, starting at 20 W (W) and increasing intensity for 10 W every minute until pulmonic or muscular exhaustion. Patients will be advised to pedal at 60 to 70 rpm. During IXT, blood pressure will be monitored using the ergometer module for automatic blood pressure management. Heart rate and cardiac rhythm will also be monitored during IXT using a 12-lead ECG (Amedtec CardioPart Blue, Amedtec, Aue, Germany). Oxygen uptake and carbon emission during IXT will be recorded using spirometry (Amedtec Ergostik, Amedtec, Aue, Germany). When patients will reach voluntary exhaustion, 20 μl of capillary blood will be withdrawn from patient’s earlobe to determine whole blood lactate concentration. Patients’ maximal performance will be defined reaching one of the following criteria: (1) capillary lactate levels of ≥8 mmol/L; (2) respiratory exchange ratio of ≥1.1; (3) maximum heart rate of 220 minus age ± 10 min^− 1^; (4) rate of perceived exertion on Borg scale ≥18 [80–82]. Incremental exercise test will be conducted under the supervision of a physician. As measures of physical fitness, patients’ maximal performance in watts per kilogram bodyweight (W/kg), as well as their peak of oxygen uptake (VO_2peak_) in milliliter oxygen uptake per kilogram bodyweight per minute, will be calculated for each participant.
- Other common cancer related side effects
  Assessment of patients’ anxiety and depression will be done according to the German version of the Hospital Anxiety and Depression Scale (HADS-D, “D” is indicating the German version of the HADS) [83]. Patients’ fatigue will be assessed using the German 20 item version of the Multidimensional Fatigue Inventory (MFI-20) [84]. Patients’ quality of sleep will be assessed using the German version of the Pittsburgh Sleep Quality Index [85]. Patients’ quality of life will be assessed by using the EORTC QLQ-C30 questionnaire and the updated breast cancer module QOL-BR23 [86]. In addition, assessment of the chemotherapy completion rates and adverse effects will be done via medical records.
- Sustainability of effects of HIIT on CRCI
  Six months after completion of the HIIT/placebo intervention (equivalent to the end of the patients’ first-line chemotherapy), the ICCTF test-battery will be additionally applied to assess the sustainability of potential positive effects of the HIIT on CRCI compared to placebo.

### Testing procedure

All assessments will take place at the Clinic Northwest in Frankfurt (Germany). In order to control for potential diurnal rhythms of biomarkers, the testing appointments will always be scheduled at 8.00 o’clock in the morning. Patients will be instructed to fast for at least 12 h before the testing appointment and to bring breakfast food along. First, an 8.5 ml fasting venous blood sample will be taken. Subsequently, patients will be given time to have breakfast. Then, patients will fill in the questionnaires (FACT-COG, EORTC QLQ-C30, QOL-BR23, PSQI, HADS-D, MFI-20). After a 15-min break, the first neuropsychological testing block comprising of the HVLT-R and the TMT will take place. It will last approximately 20 min. After another 15-min break, the second neuropsychological testing block will be conducted, applying the COWAT and the INHIB. It will take approximately 15 min. After completion of the neuropsychological testing, the IXT will be conducted lasting approximately 30 min.

### Adverse events and compliance assessment

Adverse events (AE), as well as serious adverse events (SAE), related to the exercise intervention will be assessed by the attending physicians at the Clinic Northwest in Frankfurt (Germany). Compliance to HIIT and control group intervention will be protocolled by scientific staff. It is pre-defined that patients need to participate on average in 2 exercise sessions per week following the above described treatment protocol for complete compliance. It has been shown feasible in previously published studies [51]. However, in case of emerging limitations to follow the protocol, these limitations will be addressed. The exercise regimen will be (temporarily) adapted and the patients will continue the program to the maximum extent possible.

### Data management and confidentiality

All data will be entered electronically by two independent student assistants. Any deviations between the two independent student assistants will be clarified and corrected. Original study forms will be kept on file. All study-related information will be stored in a secure manner and in accessible place. All participant information will be stored in locked in file cabinets in areas with limited access. Participant files will be kept in storage for a period of 10 years after completion of the study. Blood samples will be disposed after analyses.

All laboratory samples, reports, the data collection, the process, and all administrative forms will be identified by a coded ID number, to maintain participant confidentiality. All records that contain names or other personal identifiers, such as locator forms and informed consent forms, will be stored separately from study records, also identified by code numbers. All local databases will be secured with password-protected access systems. Forms, lists, logbooks, appointment books, and any other listings that link participant ID numbers to other identifying information, will be stored in a separate, locked file in an area with limited access. After completion of the study, raw data of all analyses will be archived in an open access repository.

### Sample size calculation

Sample size calculation was done to investigate the effect of exercise-intervention on the primary endpoint (objectively assessed cognitive performance using test battery of the ICCTF), in an analysis of covariance model (ANCOVA) using G*Power 3.1.9.2 software [87]. It was hypothesized that the changes in the test scores in the ICCTF test-battery, from baseline to after completion of the exercise-intervention, differ significantly between the HIIT group and the PCG-training group. Previous studies have shown that regular, low to moderate, exercise exerts rather small beneficial effects on CRCI in breast cancer patients [44–48]. However, pronounced effects of HIIT on fitness, cardiovascular health, fatigue and biomarkers have been reported in cancer patients [55–58]. Changes in these factors are discussed to play a pivotal role in the genesis of CRCI [24]. In accordance with it, we expect more meaningful effects of HIIT on CRCI compared to low to moderate exercise interventions. A medium effect of d = .5 was defined for sample size calculation. Alpha was set at 5%, but Bonferroni corrected due to multiple testing (4 outcome variables), leading to an adjusted alpha of 1.25%. Test power (1-β) was set at 80%. Participants’ test scores at baseline are included as covariate in the model. Required sample size in such an ANCOVA model can be calculated as (1-ρ^2^)*n, with ρ representing the correlation between participants’ baseline and post-treatment outcome-scores and n representing the sample size that would have been required, if a t-test of post-treatment outcome-scores was applied [88]. The correlation between patients’ test scores at baseline and at t_1_ was estimated with ρ = .6. This estimation was based on preliminary results (*N* = 40) of an ongoing trial of our group [89], indicating correlations between baseline and post-intervention measures of the applied tests of .6 and .8. Under the presuppositions made, the sample size calculation showed that 59 patients would be required in each group (*N* = 118). Based on attrition rate reported in existing studies applying HIIT in breast cancer patients during chemotherapy [49, 51] we accounted for 15% drop-out rate, leading to a total sample size of *N* = 136 patients.

### Statistical analysis

An intention to treat (ITT) analysis will be performed We will also assess the effect of the complete treatment in a per protocol analysis. Separate 2 × 3 mixed ANCOVAs will be conducted to determine the effects of between-subjects factor exercise-intervention, within-subjects factor measurement time-point and their interaction on primary and secondary outcomes. Patients’ baseline scores of each specific parameter will be used as covariate. A significant main effect, of within-subjects factor measurement time-point, will be further investigated through Bonferroni corrected post hoc pairwise comparisons. Simple effects analyses will be conducted to determine potential group differences at each measurement time-point.

ANCOVA assumptions will be explored. In case of violations of normality assumption, appropriate nonparametric procedures will be used. In case of violations of ANCOVA assumptions of homogenous variances or sphericity, F-test will be adjusted. Eta-square values will be reported as effect size estimates for explained variance, and Cohen’s d values will be reported for post hoc pairwise comparisons.

In addition, an exploratory regression-based mediation analysis will be performed for each intervention induced change in biomarker expression. It will be tested if the change in biomarker expression, from baseline to completion of the exercise intervention, partially mediates the effect of the exercise intervention on the change in cognitive performance. The indirect effect, as combined effect of intervention induced change in biomarker and change in biomarker induced change in cognitive performance (i.e. the effect of mediation), will be calculated (product of regression coefficients). The 95% confidence intervals will be created around the indirect effect using bootstrapping [90, 91].

Statistical analysis will be performed by a statistician blinded to treatment groups. Statistical analyses will be conducted using SPSS 25® (IBM®, Armonk, NY, USA). For mediation analyses, the PROCESS custom dialog box (version 3.1, Model 4) for SPSS [91] will be used. A result will be considered significant at *p*-value equal to or less than .5 (in case of primary endpoint this will be adjusted to .0125).

## Discussion

Despite the high incidence of CRCI among breast cancer patients, knowledge regarding effective supportive care methods is still insufficient. First results on the effects of HIIT interventions on CRCI in breast cancer are promising [49, 51], but further research is needed. The planned study will investigate the effects of a HIIT on CRCI in breast cancer patients comprehensively. The primary endpoint of the planned study will be performance in the test battery recommended by the ICCTF for investigating CRCI [59], supplemented by a classical Go-/No-Go task.

Beyond the investigation of CRCI alleviating effects of a HIIT in breast cancer patients, the present study will explore potentially underlying physiological mechanisms. To reliably attribute changes in cognitive performances, after an exercise intervention, to physiological adaptations to the exercise, exercise treatment and control group treatment must fulfill the ceteris paribus *clause*. This means that the exercise group and the control group should be equal regarding all factors except for the critical ingredient (here: the physical exertion) [92, 93]. The present study will compare the effects of a HIIT, not just against treatment as usual, but against a placebo control group. HIIT and PCG-training will both be supervised and, therefore, exhibit a comparable amount of social attention to the patients. Accordingly, it will be possible to disentangle the effects of physiological factors clearly from the potential effects of psychosocial factors related to the treatment on patients’ cognitive performances. Thus, placebo and Hawthorne effects can be minimized.

The knowledge about the immunological and neurobiological mechanisms, potentially underlying the CRCI alleviating effects of exercise, is still in its infancy. The positive impact of HIIT on inflammatory pathways, neurotrophin and growth factor expression shown in healthy individuals [57, 58], builds a strong rationale for use of HIIT in therapy of CRCI. The level of systemic inflammation, neurotrophin and growth factor expression are crucial in adult neurogenesis, survival of neurons, neurite growth, synaptic transmission and long-term potentiation [94], as well as in positive brain vasculature changes [95]. However, research on the effects of exercise on inflammatory and neurotrophic markers in breast cancer patients is still scarce. Our study will contribute to filling this knowledge gap. Several markers (TNF-α, IL-6, IL-1α, IL-1β, CRP, IL-1RA, IL-10, BDNF, IGF-1, VEGF) known to be affected by aerobic endurance training in elderly healthy individuals [40] and neurodegenerative patients [96], and believed to cause exercise-induced cognitive benefits, will be captured in the planned study. This will allow researchers to understand better the underlying mechanisms of potentially achieved training-induced cognitive benefits in breast cancer patients.

Beyond potential effects of HIIT on CRCI, HIIT might also be an especially efficient supportive therapy to alleviate other common cancer related side effects. HIIT was shown effective to increase patients’ endurance capacity [50] and, therefore, a particularly suitable method to counteract decline in physical capacity, as often experienced by breast cancer patients [97]. The effects of HIIT on other cancer related side-effects, like anxiety and depression, quality of life, sleeping disturbances, fatigue, as well as compliance to chemotherapy, remain unclear and will, therefore, also be assessed in this study.

### Trial status

The recruitment of the patients will start on 1st November, 2018. Recruitment is expected to be completed by 31st January, 2021. The data analysis and- writing of the scientific manuscripts will be carried out after completion of recruitment.

## Additional files

Additional file 1: All Items from the World Health Trial Registration Data Set. (PDF 74 kb)

Additional file 2: SPIRIT 2013 Checklist. (PDF 167 kb)

## Abbreviations

1-β: Statistical testing power
AE: Adverse events
ANCOVA: Analysis of covariance
BDNF: Brain-derived neurotrophic factor
CNS: Central nervous system
COWAT: Controlled Oral Word Association Test
CRCI: Cancer Related Cognitive Impairment
CRP: C-reactive protein
ELISA: Enzyme-linked immunosorbent assay
EORTC-QLQ-C30: Core questionnaire 30 items of the European Organization for Research and Treatment of Cancer
HADS-D: German version of the Hospital Anxiety and Depression Scale
HIIT: High-intensity interval endurance training
HR_max_: Maximum heart rate
HVLT-R: Hopkins Verbal Learning Test – Revised
ICCTF: International Cancer and Cognition Task Force
IGF-1: Insulin-like growth factor 1
IL-10: Interleukin-10
IL-1RA: Interleukin-1 receptor antagonist
IL-1α: Interleukin 1 Alpha
IL-1β: Interleukin 1 Beta
IL-6: Interleukin-6
INHIB: Response Inhibition Test
ITT: Intention-to-treat
IXT: Incremental exercise test
MFI-20: The multidimensional fatigue inventory
min: Minutes
PCG: Placebo control group
PSQI: Pittsburgh sleeping quality index
QOL-BR23: Updated breast cancer module
RCT: Randomized controlled trial
rpm: Rounds per minute
SAE: Serious adverse events
SPIRIT: Standard protocol items: recommendations for interventional trials
t_0_: Baseline measurement
-t_0_: Time-point of enrolment
t_1_: After completion of the exercise-intervention
t_2_: 6-months after cessation of patients’ first line therapy
TMT: Trail-making-test
TNF-α: Tumor necrosis factor-alpha
VEGF: Vascular endothelial growth factor
VO_2peak_: Peak of oxygen uptake in milliliter oxygen uptake per kilogram bodyweight per minute
W: Watts
W/kg: Watts per kilogram bodyweight
WTS: Wiener Test-System
