# Supplementary material for: Protocol for the “Chemobrain in Motion – study” (CIM – study): a randomized placebo-controlled trial of the impact of a high-intensity interval endurance training on cancer related cognitive impairments in women with breast cancer receiving first-line chemotherapy
Source: BMC Cancer. 2018 Nov 6;18:1071. doi: 10.1186/s12885-018-4992-3 (PMC6220507; doi:10.1186/s12885-018-4992-3)
Supplement: Supplementary file 1 — All Items from the World Health Trial Registration Data Set. (PDF 74 kb) [file 12885_2018_4992_MOESM1_ESM.pdf]

Deutsches Register Klinischer Studien
German Clinical Trials Register

Trial Description

Title

Chemobrain In Motion (CIM) - The impact of different chemotherapyaccompanying aerobic exercise programs on cancer related cognitive impairments in patients with breast cancer

Trial Acronym

CIM

URL of the trial

[---]*

Brief Summary in Lay Language

A number of cancer surivors report temporarily adverse effects in cognitive function due to chemotherapeutic treatment. Until now, there are no effective treatments to prevent these cognitive side-effects undergoing medical cancer treatment. Physical activity increasingly gains attention as a potential treatment option of CRCI. Exercise interventions have been shown to possitively influence a number of side effects and also could improve patients quality of life.
The planned study examines patients with breast cancer, respectively, shortly before highly-dose systemic chemotherapy.
Additional to their usual care treatments, the participants are randomly assigned to one of two groups. Participants assigned to the first group receive a high intensiity intervall training cycle intervention on an ergometer 3x/week for 30-35 minutes. Participants assigned to the second group receive a stretching- and mobility training for 30-35 minutes.
Before and after each training of the study, the participants perform several tests to analyse cognitive performance and thus to proof the hypothesis that endurance trainining might impact the decline of cognitive capability during chemotherapy.
Besides this main hypothesis, various aspects, such as quality of life, fatique, infection risk, psychological exposure and immune status will be documented.
(Changes to previous versions result from an amendment with the Ethics Committee of the Medical Association Hessen, March 2018)

Brief Summary in Scientific Language

The study aims to investigate the neuro-cognitive function of 130 breast cancer patients, respectively, shortly before the beginning (t0) shortly after the ending (t1) and 6 month after (t2) their highly dosed chemotherapy. A large number of cancer patients complain about cognitive impairment after implement the chemotherapy (especially verbal memory and executive function). These deficits are also called „chemobrain“ (see also Wefel & Schagen, 2012). The patients will be randomly divided in two groups; hight intensity exercise group (HIIT, EG), placebo control group (PCG). During the four weeks of highly dosed chemotherapy, participants assigned to the HIG perform a 3x/week cycling intervention on an stationary ergometer for 30-35 minutes. Participants assigned to the aKG perform a low-intensity stretching and mobilization training for the

same amount of time. The primary objective of the study is to determine if the intervention improves or maintains cognitive decline (especially verbal memory and executive function). The date will be collected per computerbased wiener testsystem (WTS). Additional, the objective measurements will be complemented by questionnaires regarding subjectivly perveived fatique, subjectivly perceived cognition performance and psychological adverse effects, caused by the cancer.

Organizational Data

- DRKS-ID: **DRKS00011390**
- Date of Registration in DRKS: **2018/01/17**
- Date of Registration in Partner Registry or other Primary Registry: **[---]***
- Investigator Sponsored/Initiated Trial (IST/IIT): **yes**
- Ethics Approval/Approval of the Ethics Committee: **Approved**
- (leading) Ethics Committee Nr.: **FF 175/2016 , Ethikkommission der Landesärztekammer Hessen**

Secondary IDs

Health condition or Problem studied

- ICD10: **C50 - Malignant neoplasm of breast**

Interventions/Observational Groups

- Arm 1: **Exercise Group: In addition to primary care via physiotherapists, patients alocated to this group will receive 30-35 minutes of a supervised high intensity training on a stationary bicycle ergometer (Ergoselect 100 Typ K, Ergoline, Bitz, Germany). Sessions will be carried out 3 times per week with at least 24 hours rest between sessions. During the first 8 weeks of intervention, training will consitst of: 5 minutes warm-up at low-intensity. Subsequently, HIIT will be conducted consisting of 3x3 min high-intensive intervals (cadence at 80-100 rounds per minutes and wattage according to 85-90% of patient`s maximum heart rate (HRmax)) with each 1.5 min in-between intervals (cadence at 60-70 rounds per minute and wattage according to 57-63% of HRmax). The last 5 minutes will be cool-down at low-intensity (cadence at 60-70 rounds per minute and wattage according to 57-63% of HRmax). During the second half of patients’ chemotherapy, the number of intervals will be increased to 5x3min.**
- Arm 2: **Placebo Control Group: In addition to standard physiotherapy participants will receive a supervised myofascial release and stretching training 3×/week for 30-35 min with at least 24 hours rest between sessions. This treatment provides an amount of social attention comparable to the Exercise Group because all myofascial release and stretching exercises will be instructed and patients will be corrected if necessary. However, unlike the ergometer training myofascial release and stretching will hardly induce**

cardiovascular arousal.

Characteristics

- Study Type: **Interventional**
- Study Type Non-Interventional: **[---]***
- Allocation: **Randomized controlled trial**
- Blinding: **[---]***
- Who is blinded: **caregiver, data analyst**
- Control: **Active control (effective treament of control group)**
- Purpose: **Supportive care**
- Assignment: **Parallel**
- Phase: **N/A**
- Off-label use (Zulassungsüberschreitende Anwendung eines Arzneimittels): **N/A**

Primary Outcome

Primary measurement is the patients outcome in several neuropsychological tests as recommended by the ICCTF, The test battery will include the Trail Making Test (TMT: selective attention, set shifting), German versions of the Hopkins Verbal Learning Test Revised (HVLT-R: verbal learning and memory) and the Controlled Oral Word Association Test (COWAT/RWT: verbal fluency). In addition, a classical Go/No-go paradigm will be used to measure executive functioning subdomain response inhibition. Measurements will take place at three points (before, shortly after and 6 month after the end of firstline chemotherapy).

Secondary Outcome

Subjective perceived impairment of cognitive performance --> FACT-COG Functional Assessment of Cancer Therapy - Cognitive Function (Version 3) and FEDA questionnaire of Experienced Deficits of Attention

Psychological stress of patients --> Hospital Anxiety and Depression Scale

Patients`fatigue --> Multidimensional Fatigue Inventory - 20 Items questionnaire

Sleep Quality --> Pittsburgh Sleeping Quality Index (PSQI)

Level of activity: Physical Activity Questionnaire (KAS, DSHS-Cologne)

Performance Status --> Classification of the Eastern Cooperative Oncology Group (ECOG Performance Status)

Vocabulary intelligence test --> MWT-B

Physical performance of patients --> spiroergometric performance

The quality of life of patients --> EORTC QLQ-C30 questionnaires

Determination of immune status and periphere neurotrophic factors of patients --> analyse relevant blood parameters (BDNF, VEGF, IGF-1), blood serum pro. (TNF-α, CRP) and anti-inflammatory cytokines (IL-1, IL-10). The analysis will be realised by using enzyme-linked immunosorbent assays (ELISAs). Measurements will take place at three points (before, shortly after and 6 month after the end of firstline chemotherapy)

Countries of recruitment

- DE **Germany**

Locations of Recruitment

- Medical Center **Krankenhaus Nordwest, Frankfurt a.M.**
- University Medical Center **Universitätsklinikum Frankfurt, Frankfurt a.M.**

Recruitment

- Planned/Actual: **Planned**
- (Anticipated or Actual) Date of First Enrollment: **2018/04/01**
- Target Sample Size: **130**
- Monocenter/Multicenter trial: **Multicenter trial**
- National/International: **National**

Inclusion Criteria

- Gender: **Female**
- Minimum Age: **18 Years**
- Maximum Age: **no maximum age**

Additional Inclusion Criteria

•Initial diagnosis of mammary carcinoma stage I-IIIA
•female
•Pending fiirst line chemotherapy (neoadjuvant or adjuvant)

Exclusion criteria

•Age < 18 years of age or > 65 years of age
•History of former cancer diagnoses
•Comorbidities that prevent participation in exercise intervention or myofascial release training (e.g. CHD, Heart failure NYHA>3, orthopedic disorders)
•Comorbidities that potentially affect cognitive functioning (e.g. psychiatric disorders, neurodegenerative diseases)

Addresses

- **Primary Sponsor**

Universitäres Centrum für Tumorerkrankungen (UCT) FrankfurtKrankenhaus NordwestKlinik für Onkologie und Hämatologie
Ms. Prof. Dr. Elke Jäger
Steinbacher Hohl 2-26
60488 Frankfurt am Main
Germany

Telephone: **069/7601-3380**
Fax: **[---]***
E-mail: **jaeger.elke at khnw.de**
URL: **[---]***

- **Contact for Scientific Queries**

Universitäres Centrum für Tumorerkrankungen (UCT) FrankfurtKrankenhaus NordwestKlinik für Onkologie und Hämatologie
Mr. M.Sc. Nils Schaffrath
Steinbacher Hohl 2-26
60488 Frankfurt am Main
Germany

Telephone: **069/7601-4522**
Fax: **[---]***
E-mail: **schaffrath.nils at khnw.de**
URL: **[---]***

- **Contact for Public Queries**

Universitäres Centrum für Tumorerkrankungen (UCT) FrankfurtKrankenhaus NordwestKlinik für Onkologie und Hämatologie
Mr. M.Sc. Nils Schaffrath
Steinbacher Hohl 2-26
60488 Frankfurt am Main
Germany

Telephone: **069/7601-4522**
Fax: **[---]***
E-mail: **schaffrath.nils at khnw.de**
URL: **[---]***

- **Collaborator, Other Address**

Institut für Kreislaufforschung und SportmedizinAbteilung für molekulare und zelluläre SportmedizinDeutsche Sporthochschule Köln
Dr. Dr. Philipp Zimmer
Am Sportpark Müngersdorf 6
50933 Köln

Collaborator, Other Address

Institut für Kreislaufforschung und SportmedizinAbteilung für molekulare und zelluläre SportmedizinDeutsche Sporthochschule Köln
Dr. Dr. Philipp Zimmer
Am Sportpark Müngersdorf 6
50933 Köln
Germany

Telephone: **0221-4982-4821**
Fax: **[---]***
E-mail: **p.zimmer at dshs-koeln.de**
URL: **[---]***

Sources of Monetary or Material Support

- **Institutional budget, no external funding (budget of sponsor/PI)**

Universitäres Centrum für Tumorerkrankungen (UCT) FrankfurtKrankenhaus NordwestKlinik für Onkologie und Hämatologie
Ms. Prof. Dr. Elke Jäger
Steinbacher Hohl 2-26
60488 Frankfurt am Main
Germany

Telephone: **069/7601-3380**
Fax: **[---]***
E-mail: **jaeger.elke at khnw.de**
URL: **[---]***

Status

- Recruitment Status: **Recruiting planned**
- Study Closing (LPLV): **[---]***

Trial Publications, Results and other documents

** This entry means the parameter is not applicable or has not been set.*
**** This entry means that data is not displayed due to insufficient data privacy clearing.*
